# Development of a measure quantifying helpful psychotherapy interventions: The Helpful Therapeutic Attitudes and Interventions Scale

**DOI:** 10.3389/fpsyt.2022.1023346

**Published:** 2022-11-22

**Authors:** Xiaobao Li, Hong Gu, Xudong Zhao, Fazhan Chen, Liang Liu

**Affiliations:** ^1^Faculty of Education, Henan University, Kaifeng, China; ^2^Clinical Research Center for Mental Disorders, Shanghai Pudong New Area Mental Health Center, School of Medicine, Tongji University, Shanghai, China

**Keywords:** psychotherapy, helpful intervention, therapeutic attitude, measure, psychometric property

## Abstract

**Background:**

Despite the number of empirical studies identifying the helpful factors correlated with beneficial psychotherapy outcomes, there is no validated scale that measures helpful therapeutic attitudes and interventions applied by therapists within the actual therapy process in China. In the current study, we developed the Helpful Therapeutic Attitudes and Interventions Scale (HTAIS) as an accessible client-rated instrument to measure useful interventions applied by the therapist during psychotherapy sessions.

**Materials and methods:**

Based on the results of previous literature and our prior psychotherapy process studies, a 40-item measure was initially designed. Two studies with a total of 2,780 Chinese participants who received psychotherapy were carried out to evaluate the structure, reliability, and validity of the developed scale.

**Results:**

Principal component analysis yielded a three-component HTAIS containing 26 items. The scale included dimensions labeled “Empathy, respect and neutrality,” “Using techniques to solve practical issues,” and “In-depth exploration and expansion.” Confirmatory factor analysis showed the construct validity of the derived three components model. And the developed scale had high internal consistency and test-retest reliability. The scale scores of the HTAIS were positively correlated with those of the Working Alliance Scale and Session Rating Scale, as well as clients’ perceived therapy satisfaction, thus supporting its concurrent validity.

**Conclusion:**

The HTAIS allows an immediate examination, as experienced by the client, of the extent to which certain useful therapeutics interventions have been conducted during therapy and could help to improve the clinician’s subsequent therapy strategies. Future research is suggested to further validate the scale, especially to assess its psychometric properties in various populations with different clinical complaints.

## Introduction

Psychotherapy is conceptualized as an approach in which trained therapists conduct professional interventions to inspire changes and improvements in the client’s perspectives, feelings, and behaviors (1). Previous literature has indicated the efficacy of psychotherapy for clients with various complaints (2, 3). However, prior studies have indicated that not all clients benefit from psychotherapy, and many factors may influence the therapy outcome (4). For the current paper, we conceptualize factors contributing to a good therapy outcome as *psychotherapy helpful factors*.

Studies that develop instruments identifying and assessing psychotherapy helpful factors have long been considered to be a central part of psychotherapy research (5, 6). Such instruments may substantially improve the design of clinicians’ daily interventions. For example, if therapists use a convenient instrument to assess the accomplished sessions and find that some helpful interventions have not been delivered, they may make synchronous and immediate adjustments to their following therapeutic strategies, such as applying more helpful therapeutic techniques and strategies. This may increase the efficiency of clients’ positive changes and the following therapy sessions, as well as reduce the time, energy, and economic costs for both therapists and clients (7).

According to a review of previous literature, we group *psychotherapy helpful factors* related to favorable therapy outcomes into two categories: *Extra-therapeutic factors* and *therapeutic factors*. *Extra-therapeutic factors* refer to those variables that occur or exist outside the actual psychotherapy process. They may consist of positive life events that increase the effect of the mechanisms of change. Such effects could be regarded as an extraneous influence on treatment outcome but not so much a consequence of treatment, unless they interact with treatment to produce the change investigated (8). *Therapeutic factors* are defined as the helpful components that occur during the actual therapy process. Implied by previous literature, *therapeutic factors* assumed to produce beneficial therapy outcomes included the *therapeutic alliance* and the *therapeutic attitudes and interventions*. Among them, the *therapeutic alliance* refers to the quality of interpersonal relationship between the clients and their therapists among therapy, including the emotional bond and the degree of agreement between the therapist and client about the tasks and goals of therapy (9). As suggested by previous research, good therapy outcomes were correlated with a high-quality working alliance between the therapist and client (5, 10–12). Concerning the instruments that assess the quality of therapist-client alliance, well-established scales include the Working Alliance Inventory (WAI) (11, 13), Working Alliance Questionnaire (WAQ) (14), and Session Rating Scale (SRS) (15). However, these measures only investigate clients’ and therapists’ experience of the quality of the therapeutic alliance. They do not measure the interventions and skills that therapists apply to build the therapy alliance.

*Therapeutic attitudes and interventions* pertain to the useful attitudes held by therapist, and the helpful techniques and interventions applied by therapists during therapy sessions. According to the previous literature, helpful therapeutic attitudes contributing to good therapy outcome include being empathetic, warm, genuineness, patient, meticulous, and amiable (7, 16). Among them, empathy refers to the therapist’s ability to enter into the subjective world of the clients and understand their feelings, experience and perceptions (17–19). It has been proved to be an important factor contributing to good therapy outcome (20). Therapeutic interventions and techniques associated with positive outcomes of therapy include transference interpretation (21), use of specific interviewing methods or procedures, use of genograms (22), use of visualization techniques (23), use of targeted homework, reformulation, use of metaphor, reflection of the therapist and client as a team, reframing (24) and use of facilitative interpersonal skills among others (25).

To date, there have been instruments purely designed to rate specific and non-specific techniques carried out by therapists during the recorded psychotherapy sessions. For example, the Collaborative Study Psychotherapy Rating Scale (CSPRS-6) is a 96-item scale measuring specific techniques of interpersonal therapy (IPT), cognitive behavioral therapy (CBT) and some non-specific therapeutic skills (26). Comparatively, the Multitheoretical List of Therapeutic Interventions (MULTI) consists of 60 items investigating key interventions from various therapy orientations, including CBT, dialectical-behavioral therapy, IPT, person-centered, psychodynamic, and process-experiential therapies (27). These observer-rated scales are used to assess how typically each psychotherapy intervention is carried out (e.g., rating the frequency and intensity of a certain technique) during the therapy. First, in comparison to client-rated scales that can be conveniently used by the clients themselves in daily clinical practice, MULTI and CSPRS-6 seem more suitable for research setting. The reason might be that, to use these instruments, the observer should accept a long-term professional training and learn how to rate the tape-recorded therapy sessions according to specific anchor points in the rater’s manual. This may restrict their popularization in daily clinical setting because most clients may not have psychotherapy training background. Second, as described above, the items in MULTI and CSPRS-6 are not specifically designed to assess whether any helpful therapy interventions are carried out by the therapist, but how typically certain techniques are used. Third, all the above scales were designed within western culture. Whether they would apply to the Chinese population is still unclear. Thus, a measure that can be conveniently used by the Chinese clients to assess the degree to which certain helpful interventions are conducted by their therapist is still needed.

The review of previous literature suggests that there are several valid instruments to measure helpful *therapeutic factors* such as the *therapeutic alliance.* Nevertheless, there is still a lack of scales that measure helpful *therapeutic attitudes and interventions* occurring during the actual therapy process in China. Duncan et al. developed the SRS to rate clients’ general satisfaction with the therapeutic relationship, clarification of goals, therapy effect and conversation style. However, the SRS did not focus on the assessment of detailed interventions carried out by therapist during treatment (15). Similarly, the Scales for the Multiperspective Assessment of General Change Mechanisms in Psychotherapy (SACiP) is an instrument measuring the subject’s experience with the treatment effect, progress and quality of the working alliance from the respective perceptions of the patient and therapist (28). However, only 3 out of 21 items (e.g., item 4, 15, and 17) in the SACiP is investigating the helpful therapeutic interventions applied by therapists during the sessions. Another measure is the Experiences of Therapy Questionnaire (ETQ) developed by Parker and colleagues. It is used to investigate clients’ impression about adverse components that contribute to unfavorable therapy outcomes. One of its dimensions pertains to the therapeutic interventions and credibility of therapists. However, it is not designed specifically for the measurement of helpful interventions applied by the therapist (29).

In China, the lack of convenient measures to assess helpful therapeutic attitudes and interventions may potentially generate two problems. First, due to a lack of immediate feedback based on reliable process measurements, therapists may continuously deliver interventions that might ultimately prove unhelpful for clients, such as overwhelming exploration of clients’ childhood trauma and negative events (30). This may lead to negative therapy outcomes and side effects for clients. Second, for trainees and supervisors of psychotherapy, it might still not be clear whether helpful therapeutic interventions have been carried out during daily therapy practice. Therefore, to address these noted gaps in the prior research, the current study aimed to (1) develop the first client-rated scale (the Helpful Therapeutic Attitudes and Interventions Scale, HTAIS) that assesses the extent to which certain helpful therapeutic interventions (which are experienced as helpful by the client) are applied by therapists during therapy in China and (2) evaluate the psychometric properties of the scale, including the test-retest reliability and validity.

Another noteworthy debate in the research of *psychotherapy helpful factor* was “whether common factors (e.g., insight, empathy, support, being genuineness) that underlie most of the psychotherapy theoretical orientations contribute more to therapy outcome than specific ingredients (e.g., transference interpretation, symbolic and analogical means of expressions such as painting or drawing in art therapy, behavioral experiment through homework assignments, and specific interviewing methods or procedures) do” (5, 6). Because previous research has not reached a unified conclusion on this topic (8), we included both some common helpful therapeutic interventions and several deliberate interventions from specific therapy orientations (as outlined in the section “Methods”) in the item selection stage.

### The present studies

The aim of present study was to develop and psychometrically validate the Helpful Therapeutic Attitudes and Interventions Scale (HTAIS). The development and psychometric evaluation of our HTAIS included two studies based on each independent sample. Study 1 aimed to develop the initial scale and to evaluate its principal components and construct validity. Study 2 assessed the concurrent validity and reliability of the HTAIS, such as the internal consistency, test-retest reliability, and subscale intercorrelations.

## Study 1 scale development and initial validation

### Methods

#### Participants and procedure

From June 15 to August 21, 2021, 2,258 individuals were recruited *via* the internet and the WeChat platform to respond anonymously to our online questionnaire if they received psychotherapy either currently or in the past. WeChat is the most representative social networking platform in China, with more than 1 billion users. The inclusion criteria included the following: (1) had received or were receiving psychotherapy; (2) had at least one therapy session in the past half year; (3) were aged 10–70; and (4) agreed to join the investigation and signed informed consent. The exclusion criteria were as follows: (1) diagnosed with severe physical disease or (2) diagnosed with severe mental disorders or mental disability without the ability to understand the content of the investigation.

The questionnaire was distributed *via* the electronic platforms of WeChat and several social psychological counseling agencies that cooperated with the authors’ institutes. When clicking on the online electronic questionnaire, participants first read a brief introduction about the aim and content of the survey and completed several items to screen their suitability for participation. Their decision to participate in the investigation and their informed consent were confirmed by clicking “yes, I agree to join this survey” before starting the questionnaire. Then, the questionnaires were completed by the participants using either a mobile app or computer interface. The completion time for the whole survey was approximately 8 min. Every participant could complete the investigation only once. After participants finished the questionnaire, they received cash compensation through the red envelope feature on WeChat. Meanwhile, participants who completed the survey were encouraged to share the investigation in their WeChat moments or to forward the survey to other WeChat groups to which they belonged. They were also encouraged to share the questionnaire with their WeChat friends who had received psychotherapy. The study was approved by the Ethics Committees of the authors’ institution.

We excluded data from 93 participants due to obviously random responses (e.g., too-short answering time and same answers for all items). Thus, the final sample 1 in study 1 consisted of 2,165 valid participants (61.7% males) aged from 10 and 66 years old (*M* = 27.39, SD = 7.75). Among the 2,165 participants, 8.7% had not completed high school, 19.7% had completed high school, 60.8% had completed college, and 10.8% of participants had received postgraduate degrees. In terms of marital status, 60.9% of participants were single, 37.7% were married, and 1.3% were divorced or widowed. The background information of participants’ participation in psychotherapy were also incorporated into the questionnaire, including their psychotherapy status (ongoing or finished), gender and age of the therapist, time of the last session, the form of therapy (individual, group, family/couple, mixture of the above forms), method of therapy (face-to-face, audio, video), venue of therapy, and whether the participants received medication. Detailed characteristics of sample 1 are listed in Table 1.

**TABLE 1:** Background information of participants’ participation in psychotherapy in sample 1.

Characteristics of psychotherapy	Options	Frequency (*N* = 2,165)	Percentage (%)
Psychotherapy status	Ongoing	1513	69.9
	Finished	652	30.1
Gender of the therapist	Male	1072	49.5
	Female	1066	49.2
Age of the therapist	Under the age of 30	445	20.6
	30∼40 years old	1111	51.3
	40∼50 years old	489	22.6
	Above 50 years old	78	3.6
	Unclear	42	1.9
When was the last time you received therapy	Nearly a week ago	636	29.4
	1 week to 1 month ago	997	46.1
	A month ago	532	24.6
Form of therapy	Individual	1158	53.5
	Group	679	31.4
	Family/Couple	192	8.9
	Mixture of the above forms	136	6.3
Method of therapy	Face-to-face	1021	47.2
	Audio	527	24.3
	Video	276	12.7
	Mixture of the above methods	309	14.3
	Other form	32	1.5
Whether the participants received medication	Yes	850	39.3
	No	1315	60.7

#### Development of the Helpful Therapeutic Attitudes and Interventions Scale

Our objective was to develop a scale that could be used conveniently to measure the helpful interventions delivered by therapists during daily psychotherapy practice. Individuals without a psychotherapy training background should be able to easily understand and respond to the items in the measure. Therefore, a client-rated scale that asked clients who received psychotherapy to rate their therapist’s behaviors was developed.

The items were designed and selected based on (1) the helpful interventions identified from our previous qualitative analysis on Chinese clients’ actual psychotherapy process. In this study, 26 h of video-recorded psychotherapy sessions from 14 Chinese cases were recruited. Thematic analysis was conducted to analyze the transcriptions of therapy sessions and 16 categories of therapy techniques, such as circular questioning and interpretation, were identified (24). In another study of ours, 12 Chinese families with depressed adolescents were interviewed after they accepted family therapy, to clarify what interventions and therapist’s attitudes they regarded as helpful. Five overarching themes related with *psychotherapy helpful factors* (e.g., facilitating emotional expression and dealing with crisis issues) were revealed with thematic analysis (7); (2) a review of previous empirical and theoretical literature focusing on psychotherapy helpful factors, clients’ subjective experiences of psychotherapy, process analysis of therapy sessions, process-outcome research, and measures on therapeutic process. A literature search was conducted in the CNKI and Web of Science databases with key search words including psychotherapy, helpful factor, process, mediator, outcome, scales and measures. Published papers, such as reviews, meta-analyses and original research, were all included.

The main helpful therapeutic attitudes and interventions were thematically extracted from previous literature and our prior research results. They included some common and theoretically specific therapy interventions. Common interventions consisted of building high-quality working alliance (11), providing empathy and acknowledgment (7, 16), enhancing resources and hope (31), promoting interpersonal meta-cognition (5), collating and classifying information (7), promoting self-insight and emotion expression (16, 32), facilitating interpersonal communication (25), promoting self-differentiation (7), and dealing with practical and crisis issues (16, 33). Specific techniques included those extracted from certain psychotherapy orientations, such as circular questioning and discussion of family interpersonal conflicts (family therapy), communication skills (interpersonal psychotherapy), behavioral experiment through homework assignments (CBT), symbolic and analogical painting or drawing (art therapy) and use of metaphor (psychodynamic and humanistic therapies) (21–24). Items were developed based on the concrete and detailed descriptions of these therapeutic interventions from the perspective of the clients. For example, concerning the intervention enhancing resource and hope, one of its related items was phrased in the following manner: “the therapist helped me (us) to see my (our) own merits, advantages and abilities.” For the therapeutic intervention promoting interpersonal meta-cognition, the item was phrased as “The therapy helped me (us) to find more positive perspectives on other people and problems.” The scale was phrased to start with the instruction: “Please, based on your actual experience, rate to what extent the following descriptions match your impression of your therapy and therapist.” Meanwhile, contents of some items from several previous scales that measure process variables such as therapy interventions and therapeutic alliance quality were also referred to (e.g., WAQ, SACiP, and CSPRS-6). For instance, the item “my counselor was receptive to even my negative thoughts and feelings” of WAQ was referred to developed item 4 “the therapist accepted my (our) emotions and thoughts” in our scale (14). Items 4 “the therapist enabled me to view my problems in new contexts” and item 17 “the therapist intentionally used the my abilities for therapy” of SACiP was taken as reference for items 21 and 26 in HTAIS, respectively (28). We also referred to the item “he/she (therapist) convey an intimate understanding of and sensitivity to the client’s experiences and feelings” of CSPRS-6 to develop item 8 “the therapist carefully observed and experienced my(our) needs and feelings” of HTAIS (26). Following this process, a 40-item self-report scale was constructed.

Participants were invited to score each item to measure the degree to which the described therapist interventions occurred throughout the actual therapy process on a 5-point Likert-type scale. The item scores ranged from 1 (not matched at all) to 5 (matched extensively). Participants were asked to share their impressions on the entire therapy process with a therapist, but not on each single session that they received (14, 34).

#### Data analysis

All statistical analyses were performed using SPSS 22.0 and Mplus 7.0 software. We mainly used principal component analysis (PCA) and confirmatory factor analysis (CFA) to verify the newly developed scale. PCA was first used to explore the underlying components of the developed scale, and CFA was used to evaluate the validity of structure obtained from PCA. To achieve cross-validation of the newly developed scale (35), the whole sample in study 1 was randomly divided into two parts: Sample A (*N* = 1083; 38.8% female; *M*age = 28.08 years, SD = 8.39) was used for PCA, whereas Sample B (*N* = 1082; 35.5% female; *M*age = 26.68 years, SD = 7.02) was used for CFA.

## Results

### Item analysis

Item analysis was used to examine the differentiability of the initial 40 items. Based on the total scores of all items, we defined the highest 27% of scores as the high-score group and the lowest 27% of scores as the low-score group. Then, multivariate analysis of variance was used to examine the differences in 40 items between groups. The multivariate tests showed that the group variable has a significant effect on items (*F* = 111.56, *p* < 0.001). And tests of between-subjects effects showed that all 40 items were significantly different between the high and low groups (the *F*-values ranged from 371.78 to 1304.99, *p*s < 0.001). In addition, all items were significantly related to the total score (the correlation coefficients ranged from 0.60 to 0.77). These results demonstrate the differentiability of all initial items.

### Principal component analysis

Principal component analysis (PCA) with varimax rotation was used to explore the best interpretable components of the developed scale. The numbers of extracted and retained components were based on eigenvalues > 1 and the Scree test. The Kaiser–Meyer–Olkin value (36) was 0.99, and Bartlett’s test was significant (χ*^2^* = 32376.52, *df* = 780, *p* < 0.001), indicating that the initial 40 items were appropriate for PCA. Regarding item selection, the remaining items were evaluated for deletion against the following criteria: (1) items that had component loadings below 0.45; (2) items that had two or more component loadings; and (3) the content contained in an item was obviously different from the other items. After repeated PCAs and item selection procedures, we obtained a 26-item scale including three components that had eigenvalues greater than 1 and that explained 63.18% of the total variance. The component loadings of the final 26 items on its components are presented in Table 2.

**TABLE 2:** Results of the principal component analysis.

Items		Component		
		1	2	3
T1	The therapist showed patience	0.67	0.17	0.20
T2	The therapist showed affinity and warmness	0.68	0.24	0.28
T3	The therapist did not force me (us) to change	0.70	0.21	0.05
T4	The therapist accepted my (our) emotions and thoughts	0.72	0.19	0.32
T5	The therapist did not judge us	0.69	0.15	0.25
T6	The therapist accepted and respected my (our) values and lifestyle	0.71	0.20	0.32
T7	The therapist talked to me (us) with equality and respect	0.73	0.15	0.34
T8	The therapist carefully observed and experienced my(our) needs and feelings	0.63	0.27	0.43
T9	The therapist respected my (our) way and speed of dealing with issues and adjusting moods	0.66	0.22	0.39
T10	The therapist gave me (us) plenty of time and opportunity to express myself (ourselves)	0.68	0.17	0.38
T11	The therapist shared some interpersonal communication skills with me (us)	0.21	0.66	0.39
T12	The therapist helped me (us) to use visual methods (for example, drawing) to present the problems	0.12	0.80	0.15
T13	The therapist helped me and my family to learn how to respect the independent space of each other	0.30	0.65	0.37
T14	The therapist asked me and my family about our perspectives on each other’s behaviors	0.29	0.67	0.31
T15	The therapist assigned me (us) interesting homework	0.19	0.81	0.17
T16	The therapist asked me (us) whether I (we) had self-injurious thoughts or behaviors and discussed with me (us) directly	0.18	0.75	0.18
T17	The therapist told some stories of others that were similar to mine (ours)	0.21	0.78	0.20
T18	The therapist discussed with me (us) about how to handle the conflicts of my (our) classmates, colleagues and friends	0.18	0.74	0.34
T19	The therapist sorted out some things and information that were originally vague and chaotic	0.43	0.28	0.61
T20	The therapist summarized the conversation in a timely manner and led us to talk more in-depth and more specifically	0.41	0.31	0.61
T21	The therapist helped me (us) to seek more perspectives on how to deal with problems and issues	0.42	0.29	0.62
T22	The therapy helped me (us) to find more positive perspectives on other people and problems	0.33	0.31	0.69
T23	The therapist helped me (us) to realize some thoughts and emotions that had not been realized before	0.40	0.25	0.64
T24	The therapist helped me (us) to see my (our) own merits, advantages and abilities	0.27	0.31	0.73
T25	The therapist encouraged me and my family to give each other the necessary support in emotion and daily life	0.29	0.41	0.63
T26	The therapist saw and affirmed my (our) abilities and advantages	0.33	0.30	0.66
% of Variance		50.78	9.20	3.98
Cumulative%		50.78	59.98	63.96

Component 1 was labeled “*Empathy, respect, and neutrality*” to reflect the therapist’s empathetic behaviors and attitudes (e.g., patience, affinity, empathy, warmth, meticulousness, professional sensitivity, and respect), as well as the therapist’s neutrality, such as not judging clients’ perspectives, values and lifestyles. It contained 10 items (T1-10 in Table 2), accounting for 50.78% of the variance in the items. The 10 items were highly loaded on this component, with coefficients ranging from 0.66 to 0.73. Component 2 was labeled “*Using techniques to solve practical issues*” to reflect that the therapist applied some specific therapeutic techniques (e.g., circular questioning, interpersonal skills, assignment of homework, and the use of drawing and metaphorical stories) to help the clients cope with practical challenges such as self-harm and interpersonal conflicts with families, classmates, colleagues and friends. There were eight items (T11-18 in Table 2) accounting for 9.20% of the variance in this factor. The loading coefficients of the eight items on this component ranged from 0.65 to 0.81. Component 3 was identified as “*In-depth exploration and expansion*” to denote the interventions that explored and expanded the client’s self-insight, multiple perspectives and resources. It consisted of therapists’ strategies that clarified complicated information, investigated clients’ unconscious processes, expanded their worldviews, facilitated clients’ self-appreciation, and encouraged clients to acknowledge the advantages and resources of themselves and their family members. It consisted of 8 items (T19-26 in Table 2) accounting for 3.98% of the variance. The loading coefficients of the 8 items on this component ranged from 0.61 to 0.73. As expected, all subscales were related to each other, with *r* values ranging from 0.52 to 0.74, and had good internal consistency (α = 0.93, 0.92, and 0.92 for component 1, component 2, and component 3, respectively; McDonald’s ω = 0.93, 0.92, and 0.92 for component 1, component 2, and component 3, respectively).

### Confirmatory factor analysis

Confirmatory factor analysis (CFA) was used to further assess the structures of the developed scale using Sample B. Before CFA, we tested the normality of the item scores. The results showed that Shapiro–Wilk values of all items ranged from 0.73 to 0.82, indicating that the data were not normally distributed. Therefore, the robust maximum likelihood (MLR) estimation robust to non-normality was used in the CFAs to yield parameter estimates (37). The comparative fit index (CFI) (≥0.95 for good, ≥0.90 for acceptable), Tucker–Lewis index (TLI) (≥0.95 for good, ≥0.90 for acceptable), root mean square error of approximation (RMSEA) (≤0.06 for good, ≤0.08 for acceptable), and standardized root mean square residual (SRMR) (≤0.08 for acceptable) were used to evaluate global model fit (38). We examined three models for different structures of the developed scale, including a single factor model (an alternative model assuming that a single latent factor represents the variation of all items), a hypothesized three-factor model, and a higher-order model (an alternative model assuming that three subscales can be regressed onto a general higher-order latent variable).

**FIGURE 1:**
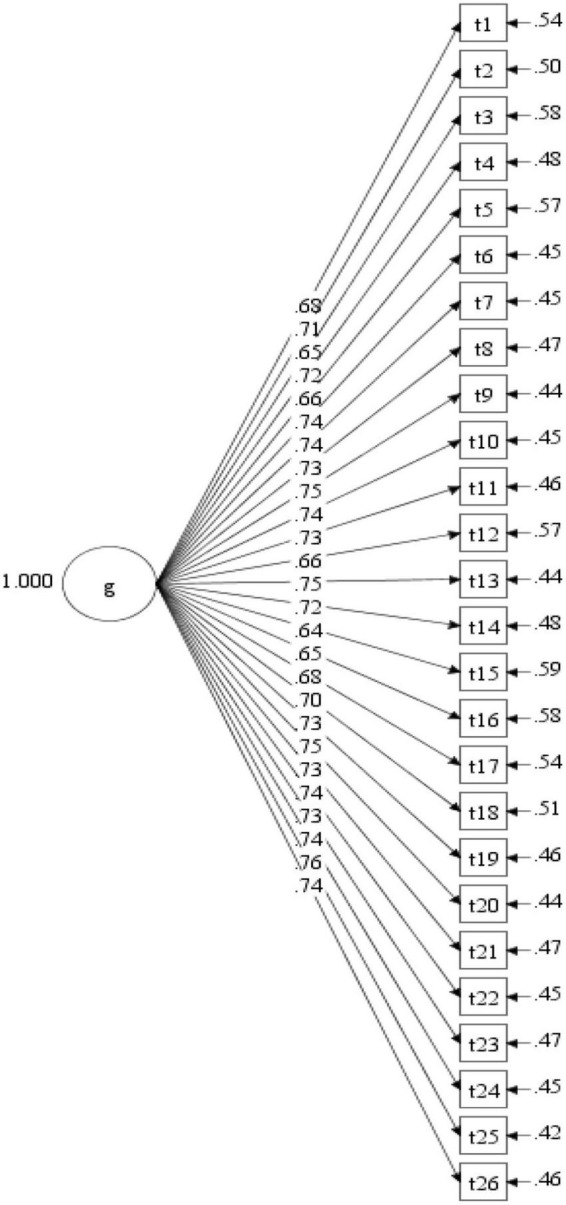
Factor loadings for the single-factor model.

**FIGURE 2:**
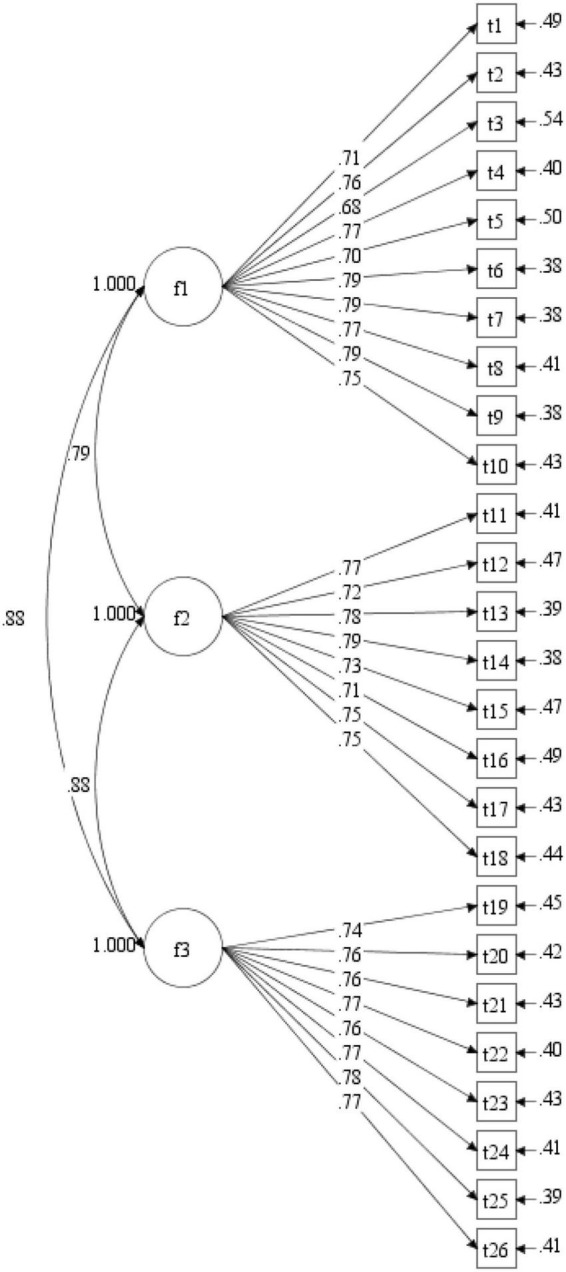
Factor loadings for the three-factor model.

**FIGURE 3:**
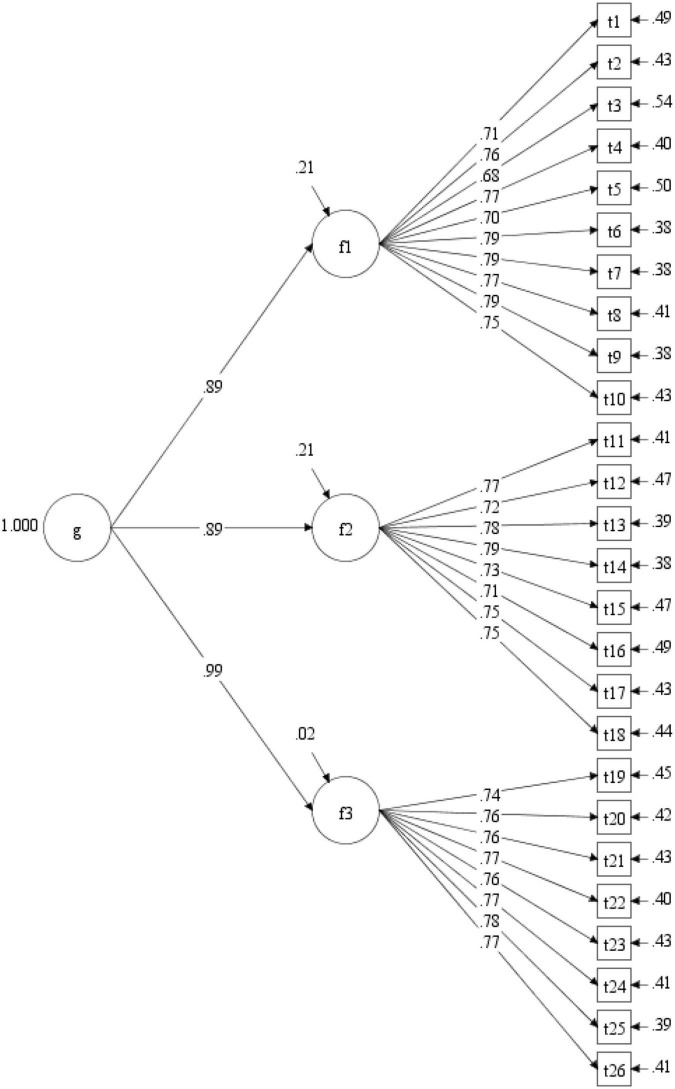
Factor loadings for the higher-order model.

The fit of the single-factor model was unsatisfactory, χ*^2^*/*df* = 4.55, CFI = 0.90, TLI = 0.89, RMSEA = 0.06, 90% CI [0.05, 0.06], SRMR = 0.05, and the factor loadings can be seen in Figure 1. Compared with that of the single factor model, the fit of the hypothesized three-factor model was significantly improved, with all fit indices reaching the ideal level, χ*^2^*/*df* = 1.97, CFI = 0.97, TLI = 0.97, RMSEA = 0.02, 90% CI [0.03.03], SRMR = 0.03. The factor loadings of all items loaded on their factors ranged from 0.68 to 0.79, all of which were beyond the critical value of.40 (see Figure 2). The fit of the higher-order model (χ*^2^*/*df* = 1.97, CFI = 0.97, TLI = 0.97, RMSEA = 0.03, 90% CI [0.03.03], SRMR = 0.03) was nearly equivalent to that of the three-factor model, and all values of fit indices were the same, except that the RMSEA of the higher-order model was slightly higher than that of the three-factor model. Figure 3 shows the factor loadings for the higher-order model. The good fit of the three-factor and higher-order model supports the three-factor structure of the developed scale and the possibility of creating a total scale composite score for use in future research.

## Study 2 reliability and validity

### Methods

#### Participants and procedure

From September 2 to November 17, 2021, the questionnaire was distributed *via* the WeChat platform and the counseling centers of 4 universities in Shanghai, China. A total of 647 participants who had finished or were currently undergoing psychotherapy were recruited. The inclusion and exclusion criteria were the same as those in study 1. All participants consented to participate in the study and signed the informed consent form. The investigation obtained approval from the local ethics committee. It took approximately 10∼15 min to complete several scales, including the 26-item HTAIS, the SRS, the WAQ, and a scale on psychotherapy satisfaction. Data from 32 participants were excluded because of missing responses or obviously random responses.

A final sample of 615 participants was obtained in study 2. The age range of the participants was 12∼67 years old (*M* = 30.46, SD = 10.71), and 18.5% were males. Among the 615 participants, 60% were single, 36.3% were married, and 3.7% were divorced or widowed. Regarding education information, 37.4% of participants had received postgraduate degrees, 55.6% had completed college, 4.6% had graduated from high school, and 2.4% reported they had not completed high school. The background information of participants’ participation in psychotherapy, as in study 1, were also included in the questionnaire. Detailed characteristics of sample 2 are listed in Table 3.

**TABLE 3:** Background information of participants’ participation in psychotherapy in sample 2.

Characteristics of psychotherapy	Options	Frequency (*N* = 615)	Percentage (%)
Psychotherapy status	Ongoing	424	68.9
	Finished	191	31.1
Gender of the therapist	Male	144	23.4
	Female	471	76.6
Age of the therapist	Under the age of 30	43	7.0
	30∼40 years old	252	41.0
	40∼50 years old	195	31.7
	Above 50 years old	58	9.4
	Unclear	67	10.9
When was the last time you received therapy	Nearly a week ago	344	55.9
	1 week to 1 month ago	94	15.3
	A month ago	177	28.8
Form of therapy	Individual	542	88.1
	Family/Couple	35	5.7
	Mixture of the above forms	38	6.2
Method of therapy	Face-to-face	542	88.1
	Audio or video	35	5.7
	Mixture of the above methods	38	6.2

In addition, we selected a subset of participants from sample 2 to complete the HTAIS again at 2 weeks apart for examining test–retest reliability. These participants were reminded to again complete HTAIS *via* e-mail. A total of 134 participants (9.7% males, *M* = 37.42, SD = 9.42) from sample 2 who completed the repeated measure were included for analysis.

### Measures

#### Helpful Therapeutic Attitudes and Interventions Scale

All participants completed the HTAIS developed in Study 1. In the present sample, the Cronbach’s α coefficients for *Empathy, respect, and neutrality*, *In-depth exploration and expansion*, *Using techniques to solve practical issues*, and *the total scale* were 0.94, 0.93, 0.90, and 0.95, respectively. The McDonald’s ω for the three factors were 0.94, 93, 0.89, and 0.94, respectively.

#### Session rating scale

The Chinese version of the SRS was used to assess the therapeutic alliance between the therapist and client (15). This scale has 4 items and measures the views of clients toward their therapists, agreement on goals and topics, methods, and overall experience. All items were rated on a 10-cm line with markers from left (negative) to right (positive). A higher total score of four items indicates a better working alliance between therapists and clients. In the present study, the Cronbach’s α coefficient of this scale was 0.90. The McDonald’s ω for this scale was 0.90.

#### Working alliance questionnaire

The WAQ was used to assess the quality of the cooperative relationship between clients and therapists (14). This scale has 12 items and three dimensions: relationship bond, goal-task, and client engagement. All items are rated on a 1 (rarely) ∼ 5 (always) Likert scale. A higher total score reflects an increased level of therapeutic alliance. In the present study, the Cronbach’s α coefficients for relationship bond, goal-task, client engagement, and the total scale were 0.88, 0.87, 0.85, and 0.94, respectively. The McDonald’s ω for the three factors and total scale were 0.87, 0.87, 0.85, and 0.94, respectively.

#### Psychotherapy satisfaction

At the end of our investigation, participants were also asked to rate the level to which they perceived the psychotherapy to be satisfactory on a scale ranging from 1 (not satisfied) to 10 (very satisfied). In the present study, the mean scores of psychotherapy satisfaction were 8.47 (SD = 1.40).

### Data analysis

All statistical analyses were performed using SPSS 22.0 software. Internal consistency was assessed using McDonald’s ω. Test-retest reliability was assessed using Pearson correlations between the developed scale scores at time 1 and at time 2. Concurrent validity was examined using Pearson correlations between the developed scale and the SRS, the WAQ, and the scale on self-rated satisfaction with psychotherapy.

## Results

### Reliability and interdimensional correlations

Means, standard deviations, internal consistency, test-retest reliability, and intercorrelations of the developed scale are presented in Table 4. Based on sample 2, we calculated the Cronbach’s alpha to determine the internal consistency of the developed scale. The results showed that *Empathy, respect, and neutrality*, *In-depth exploration and expansion*, *Using techniques to solve practical issues*, and *the total scale* had good internal consistencies, with McDonald’s ω = 0.94, 93, 0.90, and 0.94, respectively. Meanwhile, all three subscales and the total scale had adequate test-retest reliability, with coefficients ranging from 0.68 to 0.81. Regarding the intercorrelations, we found that all three subscales were positively correlated with each other at a moderate to high level.

**TABLE 4:** Reliability and interdimensional correlations of HTAIS.

	*M*	SD	ω	Test-retest reliability	1	2	3
1. Empathy, respect, and neutrality	4.54	0.56	0.94	0.68	1		
2. In-depth exploration and expansion	4.33	0.67	0.93	0.68	0.71**	1	
3. Using techniques to solve practical issues	3.54	0.97	0.90	0.81	0.41**	0.65**	1
Total scale	4.14	0.63	0.95	0.77	0.77**	0.90**	0.87**

**p < 0.01.

### Concurrent validity

Concurrent validity was assessed by examining Pearson correlations between the HTAIS and the WAQ, the SRS, and psychotherapy satisfaction. Table 5 shows the correlation coefficients between the HTAIS and the other constructs. The results showed that *Empathy, respect, and neutrality*, *Using techniques to solve practical issues*, *In-depth exploration and expansion* and the total scores were all positively correlated with the WAQ and SRS. Regarding the self-rated outcomes about psychotherapy, the overall scale and its three subscale scores all had significant positive correlations with psychotherapy satisfaction. In other words, when psychotherapists had higher scores on the overall HTAIS, the clients reported greater satisfaction with the therapy. These results support the concurrent validity of our measure.

**TABLE 5:** Concurrent validities of HTAIS.

	*M*	SD	Empathy, respect and neutrality	In-depth exploration and expansion	Using techniques to solve practical issues	Total scale
WAQ	6.25	1.13	0.62**	0.75**	0.56**	0.74**
Goal-task	4.04	0.83	0.53**	0.74**	0.60**	0.73**
Bond	4.29	0.77	0.68**	0.69**	0.46**	0.69**
Engagement	4.18	0.84	0.51**	0.65**	0.49**	0.64**
SRS	34.18	5.64	0.60**	0.67**	0.44**	0.64**
Psychotherapy satisfaction	8.47	1.40	0.59**	0.70**	0.49**	0.67**

**p < 0.01.

## Discussion

The objective of the current study was to develop a convenient measure designed to investigate helpful therapeutic attitudes and interventions conducted by psychotherapists during daily practice and test its psychometric properties. An initial version of the scale containing 40 items was developed. After item reduction and CFA, a three-component scale with 26 items that met the criteria for interpretability was retained. The internal consistency of the HTAIS was good (with McDonald’s ω coefficients of the subscales ranging from 0.90 to 0.94 and a coefficient of 0.95 for the total scale). Meanwhile, correlations between the subscale scores were moderate and significant (correlation coefficients 0.41–0.71), hence demonstrating that the factors are distinct but related. Test-retest reliability was assessed by applying the HTAIS to some of the participants after 2 weeks. The results showed significant and acceptable correlation coefficients (0.68–0.80), indicating satisfactory temporal reliability of the retained factors.

A review of the retained scale items implied that both some common interventions underlying most psychotherapy theories (e.g., showing empathy) and a few deliberate skills of specific therapy orientations (e.g., circular questioning and metaphor) were identified as helpful by the clients. Regarding *Empathy, respect, and neutrality*, our findings demonstrated that therapists’ attitude toward humanism was one of the important factors promoting positive outcomes. This coincides with previous research indicating that therapists’ humanistic traits could facilitate the establishment of trustworthy therapist-client relationships, which may consequently contribute to beneficial therapy changes (5, 7, 16). With respect to *In-depth exploration and expansion*, therapist behaviors such as promoting clients’ self-insight and investigating unconscious processes have been proven helpful by previous psychodynamic research (5, 16, 39). Comparatively, items related to the expansion of clients’ worldviews were identified as commonly used helpful strategies in CBT and systemic therapy (24, 40). Meanwhile, from the clients’ perspective, achieving self-insight, gaining multiple perspectives and attaining psychological resilience were regarded as belonging to the same group of techniques. This finding implied that clients may consider these three kinds of interventions to be helpful factors closely correlated with each other and co-contributing to positive outcomes. This is partially consistent with the existing findings that clients expected that changes, including self-insight, cognitive adjustment and attainment of confidence, could be achieved simultaneously during psychotherapy (5). *Using techniques to solve practical issues* focused on the process by which therapists conducted deliberated techniques to facilitate clients’ solutions to actual stress and challenges. This finding coincides with prior research suggesting the necessity of incorporating targeted therapeutic strategies for the solution of daily issues and crises in psychotherapy (7, 16). More importantly, the three factors developed in this study match the three-stage helping skills model (exploration, insight or understanding, and action) used for therapists training (41). For instance, *In-depth exploration and expansion* can be used to assess therapists’ helping skills of exploring clients’ specific problems. *Empathy, respect, and neutrality* can be used to assess therapists’ attitudes of understanding or showing respect to clients’ problems. And *Using techniques to solve practical issues* assess therapists’ actions on assisting clients to change their feelings, thoughts, and behaviors. Thus, using HTAIS might get a clearer picture about the level of working relationship between therapists and clients through the evaluation of therapists’ three-stage helping skills from client perceptions.

Another interesting finding of our study was that participants were more impressed by “warm, easy-to-understand, visible, and practical” therapeutic strategies (e.g., showing empathy and respect, drawing, discussing how to solve family conflicts) than by certain “profound and professional” theoretical approaches, such as the promotion of self-differentiation (7) and psychodynamic interpretation (21). For example, two items related to psychodynamic interpretation (“the therapist analyzed the reasons for my/our problems” and “the therapist provided professional explanations and knowledge for my/our confusion”) and another self-differentiation item (“the therapist encouraged my family to support each other’s self-development”) were included in the initial version of our scale. However, the results of the factor analysis did not support retaining these items. This implies that clients might be generally insensitive to the theoretical mechanisms underlying different “dialog therapies,” such as psychoanalysis or family therapy (5, 6).

The concurrent validity of the HTAIS was assessed by examining the associations of the HTAIS with the WAQ and the SRS. Significant and moderate correlations were found between all HTAIS subscales and every dimension of the WAQ and SRS. This implies that the concurrent validity is acceptable. The WAQ was designed to investigate Chinese clients’ experience with the quality of the working alliance established with the therapist. It consists of three components: *bond, goal-task* and *engagement* (14). The results of correlation analysis implied that, although all correlations were significant, the *Empathy, respect, and neutrality* dimension of the HTAIS was slightly more correlated with the *bond* dimension of the WAQ than to its other two dimensions. This finding seems reasonable because previous literature indicated that therapists’ humanity and respect were important contributors to the establishment of a trustworthy therapist-client relationship (6, 42). Regarding the *In-depth exploration and expansion* dimension of the HTAIS, its correlation coefficients with the three dimensions of the WAQ were all within similar ranges (0.65–0.74). A possible explanation might be that the exploration of clients’ resources and multiple perceptions may inspire their motivation, enthusiasm and confidence for change. This may promote their *engagement* in therapy, as well as their *emotional bond* with the therapist. Meanwhile, the clarification of complex information and promotion of self-insight may help clients be more aware of their expectations of their *therapeutic tasks* and life goals. Again, our findings implied that self-insight, changes in perception and the attainment of psychological resilience may be considered by clients as interventions closely correlated with each other (5, 6). Concerning the factor *Using techniques to solve practical issues*, its correlation coefficient with the *goal-task* dimension of the WAQ was slightly higher than those with *bond* and *engagement.* The reason for this discrepancy may be that *goal tasks* in WAQ reflect more about “the achievement of visible changes,” which coincides with the content of *Using techniques to solve practical issues* in the HTAIS (14).

Additionally, the three factors and the total score of the HTAIS were found to be positively correlated with perceived psychotherapy satisfaction. This finding suggested that the derived components of the HTAIS were helpful interventions related to the perception of a positive outcome (5, 30). Comparatively, our analysis demonstrated that the HTAIS factor *In-depth exploration and expansion* was more correlated with all the subscales and total score of WAQ, perceived psychotherapy satisfaction, as well as the SRS, which was designed to measure client’s general satisfaction with therapy process, working alliance and achievement of therapy goal. This implied that *In-depth exploration and expansion* might be considered a more important component related to the expected outcome. The reason may be that this factor contained more helpful factors, as proven by previous literature (5, 6, 16). However, more research is still suggested to explore the discrepancies among the relationships between these measures and HTAIS interventions, as well as the mechanism by which these interventions co-contribute to a beneficial therapy outcome.

There are a number of limitations in the present study. First, we developed a measure investigating clients’ impression of the entire therapy process. However, psychotherapy is a dynamic process characterized by constant changes, including the therapist’s behaviors. Hence, future research to design more sophisticated scales assessing therapists’ helpful interventions during each single session (at the session level) is strongly suggested (34). Second, although HTAIS consists of both common therapeutic factors and interventions from specific therapy orientations, the choice for the included therapeutic interventions might also be a limitation. It cannot assess the helpful factors of every independent therapy orientation. This may restrict its popularization and implementation in some professional training and clinical programs that emphasize the development of competence belonging to certain therapy theories. Future research can be conducted to develop a helpful therapy attitudes and interventions scale for each specific therapy modality. Third, most of the participants in sample 2 were female. Although there were still 114 male participants (18.5%), which guaranteed the statistical validity of our analysis, this imbalance in the sex ratio might still have introduced bias to the results. In addition, we did not collect the therapeutic approaches received by clients in our data set, which is not conducive to compare the effects of different therapeutic approaches on the clients’ evaluation of therapists’ attitudes and interventions. Thus, future investigation including more male participants and distinguishing therapeutic approaches is suggested. Fourth, the majority of the client feedback in our study was collected through the internet. Although convenient, this data recruitment strategy might also reduce the quality and reliability of the data. In our future research, more face-to-face data recruitment will be conducted. Fifth, we measured only clients’ perceptions about helpful therapy interventions without designing a corresponding version for the therapist perspective. As suggested by previous research, the experience of therapists and clients with the therapy process may be different (28). A comparison between the perceptions of therapists and clients may help improve intervention strategies and enhance therapy relationships and positive outcomes (7). Hence, a therapist version of a helpful therapy attitudes and interventions scale should be developed in future studies, and the discrepancies and synchronization between therapists’ and clients’ ratings should also be explored. Sixth, although the interventions of the HTAIS were found to be positively correlated with perceived satisfaction with psychotherapy, more empirical research is still needed to explore the relationships between HTAIS ratings and the actual outcomes of psychotherapy such as the relief of symptoms and improvement in client’s social function.

## Conclusion

In the current study, we developed the HTAIS as an instrument designed to measure helpful therapy interventions (which are experienced as helpful by the client) delivered by therapists during psychotherapy. An initial evaluation of the psychometric property of the HTAIS demonstrated that it was a reliable and valid measure. The exploration of the identified constructs yielded three components to reflect therapists’ empathetic interventions, expansion of self-insight and resources, and treatment of practical issues using deliberate skills. The HTAIS could be distributed to clients after therapy to collect their immediate comments and to reflect on whether necessary helpful interventions have been conducted within the accomplished therapy. This could help to improve the clinician’s subsequent therapy strategies. Future research is suggested to further validate the scale, especially to assess its psychometric properties in various populations with different clinical complaints. Such a series of studies can advance the understanding of the relationship between therapists’ therapeutic interventions and expected therapy outcomes, as well as the mechanisms by which psychotherapy works.

## Data availability statement

The raw data supporting the conclusions of this article will be made available by the corresponding author, without undue reservation.

## Ethics statement

The studies involving human participants were reviewed and approved by the Ethics Committee of Tongji University as well as the Shanghai Pudong New Area Mental Health Center (No. PWRd2020-01). Written informed consent to participate in this study was provided by the participants’ legal guardian/next of kin.

## Author contributions

LL and XL made substantial contributions to the design, data analysis, and manuscript draft. LL, FC, and HG contributed to the participants and data recruitment. XZ contributed to the study conception and design. All authors read and approved the final manuscript.
